# Strahleninduzierter Katarakt – ein okkultes Berufsrisiko für Urologen

**DOI:** 10.1007/s00120-023-02073-w

**Published:** 2023-05-03

**Authors:** J. J. Wendler, J. Schittko, A. Lux, U.-B. Liehr, M. Pech, M. Schostak, M. Porsch

**Affiliations:** 1 Klinik für Urologie, Uroonkologie, robotergestützte und fokale Therapie, Medizinische Fakultät, Otto-von-Guericke-Universität Magdeburg, Leipziger Straße 44, 39120 Magdeburg, Deutschland; 2 Praxis für Urologie, BAG, Magdeburg, Deutschland; 3 Institut für Biometrie, Universitätsklinikum Magdeburg A.ö.R., Magdeburg, Deutschland; 4 Klinik für Radiologie und Nuklearmedizin, Universitätsklinikum Magdeburg A.ö.R., Magdeburg, Deutschland

**Keywords:** Strahlenschutz, Urologie, Jahresgrenzdosis, Augenlinse, Strahlenschaden, Radiological protection, Urology, Annual dose limit, Eye lens diseases, Radiation injury

## Abstract

**Hintergrund:**

Der Strahlenkatarakt stellt ein relevantes Risiko für beruflich strahlenexponierte Personen dar. Die Jahresgrenzdosis für die Augenlinse wurde auf 20 mSv pro Jahr per Gesetzgebung (Strahlenschutzgesetz StrlSchG 2017; 2013/59/Euratom) nach Empfehlung der Internationalen Strahlenschutzkommission (2011 ICRP) zur Vermeidung eines strahleninduzierten Katarakts gesenkt.

**Fragestellung:**

Besteht die Gefahr der Überschreitung der Jahresgrenzdosis für die Augenlinse in der urologischen Routine ohne spezielle Strahlenschutzmaßnahmen für den Kopf?

**Methodik:**

Im Rahmen einer prospektiven, monozentrischen Dosimetriestudie von 542 verschiedenen urologischen, fluoroskopisch geführten Interventionen erfolgte die Bestimmung der Augenlinsendosis per Stirndosimeter (Thermolumineszenz-Dosimeter TLD, Chipstrate) über einen Zeitraum von 5 Monaten.

**Ergebnisse:**

Es zeigte sich eine durchschnittliche Kopfdosis von 0,05 mSv pro Intervention (maximal 0,29 mSv) bei einem durchschnittlichen Dosisflächenprodukt (DFP) von 485,33 (21,7–3731,2) Gy/cm^2^. Signifikante Einflussfaktoren auf eine höhere Dosis waren ein höherer BMI des Patienten, eine längere Operationsdauer und ein höheres DFP. Das Erfahrungslevel des Operateurs zeigte keinen signifikanten Einfluss.

**Diskussion:**

Mit 400 Eingriffen pro Jahr oder durchschnittlich 2 Eingriffen pro Arbeitstag wäre damit der kritische Jahresgrenzwert für die Augenlinsen bzw. für das Risiko eines Strahlenkatarakts ohne spezielle Schutzmaßnahmen überschritten.

**Schlussfolgerung:**

Ein konsequenter effektiver Strahlenschutz der Augenlinse ist essentiell für die tägliche Arbeit bei uroradiologischen Interventionen. Hierfür sind ggf. technische Weiterentwicklungen erforderlich.

## Abstract (en)

**Background:**

Radiation-induced cataracts represent a relevant risk to people occupationally exposed to radiation. The annual limit dose for the eye lens was reduced to 20 mSv per year by German legislation (radiation protect law StrlSchG 2017; 2013/59/Euratom) based on recommendation of International Commission on Radiation Protection (2011 ICRP) to avoid radiation-induced cataracts.

**Objectives:**

Is there a risk of exceeding the annual limit dose for the eye lens in routine urological practice without special radiation protection for the head?

**Methods:**

As part of a prospective, monocentric dosimetry study, of 542 different urological, fluoroscopically guided interventions, the eye lens dose was determined using a forehead dosimeter (thermo-luminescence dosemeter TLD, Chipstrate) over a period of 5 months.

**Results:**

An average head dose of 0.05 mSv per intervention (max. 0.29 mSv) was found with an average dose area product of 485.33 Gy*/*cm^2^. Significant influencing factors for a higher dose were a higher patient body mass index (BMI), a longer operation time, and a higher dose area product. The level of experience of the surgeon showed no significant influence.

**Discussion:**

With 400 procedures per year or an average of 2 procedures per working day, the critical annual limit value for the eye lenses or for the risk of radiation-induced cataract would be exceeded without special protective measures.

**Conclusion:**

Consistently effective radiation protection of the eye lens is essential for daily work in uroradiological interventions. This may require further technical developments.

Der Strahlenkatarakt stellt ein relevantes Risiko für beruflich strahlenexponierte Personen dar. Die Jahresgrenzdosis für die Augenlinse wurde auf 20 mSv pro Jahr per Richtlinien (Strahlenschutzgesetz StrlSchG 2017; 2013/59/Euratom) nach Empfehlung der Internationalen Strahlenschutzkommission (2011 ICRP) zur Vermeidung eines strahleninduzierten Katarakts gesenkt. Insbesondere interventionell und operativ tätige Urologen sind einer erhöhten Augenlinsendosis während fluoroskopischen Operationen ausgesetzt. Ohne einen speziellen Strahlenschutz für die Augen kann die Jahresgrenzdosis schnell überschritten werden.

## Einleitung

**Figure 1:**
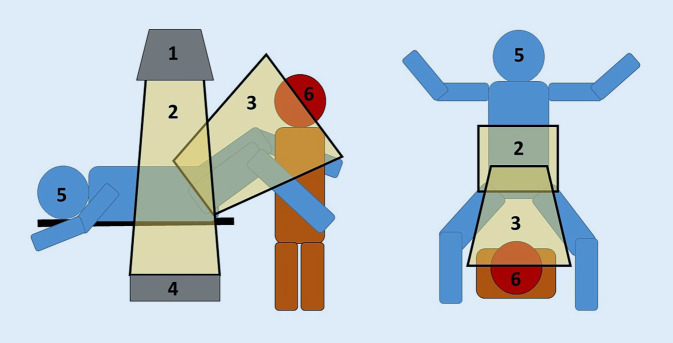

Die Jahresgrenzdosis für die Augenlinse beruflich strahlenexponierter Personen wurde gesetzlich von 150 auf 20 mSv pro Jahr durch das Bundesministerium für Umwelt, Naturschutz, nukleare Sicherheit und Verbraucherschutz (StrlSchG 2017) sowie durch den Rat der Europäischen Union (RL 2013/59/Euratom) nach Empfehlung der Internationalen Strahlenschutzkommission (ICRP 2011) zur Vermeidung eines strahleninduzierten Katarakts festgelegt [1–3]. Insbesondere Urologen sind einer erhöhten Augenlinsendosis (ALD) während fluoroskopischen Operationen ausgesetzt. Der zunehmende Anteil der endourologisch-interventionellen, minimal-invasiven Eingriffe sowie der Patienten mit Adipositas bewirken eine Zunahme der Strahlenexposition der Operateure. Diese befinden sich bei transurethralen Operationen in Steinschnittlage zwischen den angewinkelten Beinen des Patienten, sodass der frontal zur Röntgenröhre gerichtete und schräg oberhalb des Beckens befindliche Kopf unmittelbar dem Hauptteil der Streustrahlung ausgesetzt ist (Abb. 1).

Durch den begrenzten Platz im sterilen Arbeitsbereich können bauliche Strahlenschutzmaßnahmen wie mobile Bleiglasschilde nicht bzw. kaum eingesetzt werden. Strahlenschutzvisiere sind nicht arbeitsmedizinisch bzw. strahlenschutztechnisch gefordert und nicht flächendeckend etabliert, da sie oft aus Komfortgründen vom Operateur abgelehnt werden [4]. Röntgenschutzbrillen sind in der Regel vorhanden, werden aber auch oft aus Komfortgründen und fehlender Sensibilisierung des Personals nicht getragen. Weiterhin kommen verschiedene Laser bei einem Großteil der endourologischen Eingriffe unter fluoroskopischer Kontrolle zur Anwendung, wobei eine Kombination aus Laserschutzbrille und Röntgenschutzbrille nicht zur Verfügung steht. Bekannte Daten zur Augenlinsendosis des Operateurs im Rahmen interventionell-urologischer Eingriffe sind aufgrund von unterschiedlichen Untersuchungsmethoden jedoch nicht allgemein übertragbar [5–11]. Strahlendosen sind zwar in der interventionellen Radiologie und Kardiologie hinreichend untersucht. Im Vergleich konnten z. B. Vano et al. jedoch zeigen, dass Urologen einer 18,7-mal höheren Strahlenbelastung als interventionell tätige Kardiologen mit entsprechenden Strahlenschutzmaßnahmen wie Bleiglasschilden und einer 4,2-mal höheren Strahlenbelastung als Gefäßchirurgen ausgesetzt waren [12]. Mit unserer Arbeit wollten wir die Augenlinsendosis der Operateure pro Intervention in Abhängigkeit verschiedener möglicher Einflussfaktoren in der klinischen Routine mit modernen urologischen Röntgentischanlagen und ohne spezielle Strahlenschutzmaßnahmen für den Kopf untersuchen und ob eine Gefahr der Überschreitung der neuen Jahresgrenzdosis für die Augenlinse von 20 mSv und damit das Risiko eines Strahlenkatarakts besteht [13].

## Methodik

**Figure 2:**
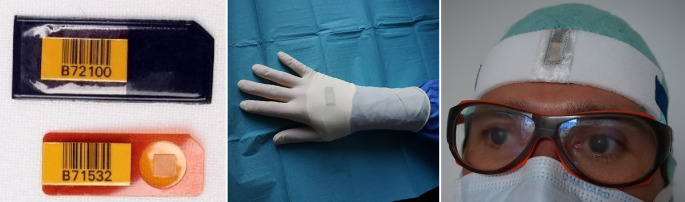

Die Bestimmung der Augenlinsendosis erfolgte im Rahmen einer nicht genehmigungspflichtigen, zweiphasigen, prospektiven, monozentrischen Beobachtungsstudie zur Teilkörperdosismessung im Rahmen uroradiologischer Routineeingriffe im universitär-klinischen Setting („multi-surgeon, single-institute“) gemäß § 76 Abs. 1 Satz 10 Strahlenschutzgesetz. Zunächst erfolgte in der ersten Phase die kumulative Handdosismessung mittels Ringdosismeter (Landesanstalt für Personendosimetrie und Strahlenschutzausbildung LPS, Berlin, BRD; Harshaw TLD^TM^  Thermo Fisher Scientific, MA, USA) monatlich über einen Zeitraum von 12 Monaten über 875 Interventionen, wobei keine Aussagen über die Strahlendosis pro Eingriff getroffen werden konnte. In der zweiten Phase erfolgte die Bestimmung der Teilkörperdosen von Hand und Kopf mittels Thermolumineszenzdosimeter (TLD) vom Typ XD-700 (Landesanstalt für Personendosimetrie und Strahlenschutzausbildung LPS, Berlin, BRD; Harshaw TLD^TM^ Thermo Fisher Scientific, MA, USA) in Form von Chipstrate-Dosimetern an Handrücken (Führungshand) und Stirn (mediofrontal mittels Stirnband) repräsentativ für die Augenlinsen der Operateure (Abb. 2) über einen Zeitraum von 5 Monaten.

**Figure 3:**
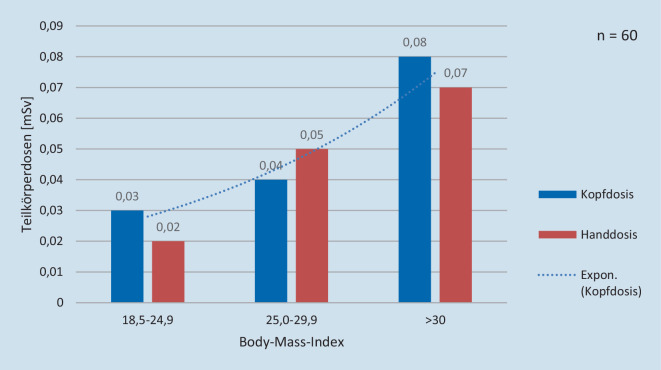

Die TLD wurden pro Eingriff von der Landesanstalt für Personendosimetrie und Strahlenschutzausbildung (LPS, Berlin) mittels des automatischen TLD-Reader HARSHAW 6600 CCD ausgelesen. Ein Nulldosimeter pro verwendeter Charge verblieb im Überwachungsraum, um die Hintergrundstrahlung von den Messwerten zu subtrahieren. Die Dosimetersonde besteht aus zwei Teilen: einer Einweghülle (Kunststoffpouch) und dem TLD auf einem rechteckigen Kaptonstreifen mit Barcode. Der Detektor selbst besteht aus einer quadratischen TLD-Tablette (3,175 × 3,175 mm, 0,381 mm dick) aus Lithiumfluorid (Nuklid Li‑7; [14]). Die Chipstrates wurden auf dem Quaderphantom (Strahlenqualität A80) in der Messgröße Oberflächenpersonendosis Hp(0,07) kalibriert. Die desinfizierbaren und sterilisierbaren Dosimeter erfassen Photonenstrahlung im Energiebereich von 15–1250 keV und Dosen von 0,05–1000 mSv unter einer Strahleneinfallsrichtung von ±60° [14]. Alle Interventionen wurden mit dem Röntgensystem Uroskop Omnia (Siemens Healthcare Solutions, Erlangen) unter gepulster Durchleuchtung (Pulsfrequenzen von 3;7,5;10 P/s) und mit „Last image hold“-Funktion durchgeführt. Dieses flachdetektorbasierte Röntgensystem besteht aus einem Obertischröntgenstrahler (Poyldoros F, Optitop 150/40/80 HC-100, Aufnahmespannung 40–150 kV) mit Tiefenblende, Belichtungsautomatik und einem Untertischbildverstärker. Bei dem verwendeten Röntgendetektor handelt es sich um einen amorphen Siliziumflachdetektor mit einer räumlichen Auflösung von 3,4Lp/mm bei einer Pixelgröße von 148 um und einer maximalen Aufnahmegeschwindigkeit von bis zu 8 bzw. 15 Bilder pro Sekunde im Durchleuchtungsmodus. Als Parameter wurden die Patientendaten (Diagnose, Körpergewicht KG, Body Mass Index/BMI), die Operationsart (Operationstechnik, Operationsdauer), Positionen der Operateure zu den Patienten und Patientinnen sowie zur Röntgenröhre (Abb. 2), das Dosisflächenprodukt (DFP) sowie das Erfahrungslevel der Operateure dokumentiert. Die Einteilung der Operateure nach Erfahrungslevel erfolgte in 3 Gruppen: Level 1: Assistenzärzte < 3 Jahre Berufserfahrung, Level 2: Assistenzärzte bzw. Fachärzte mit 3–6 Jahren Berufserfahrung; Level 3: Oberärzte mit > 6 Jahren Berufserfahrung.

## Ergebnisse

Im Rahmen der interventionsbezogenen Teilkörper-TLD-Dosimetrieuntersuchung wurden bei 60 auswertbaren uroradiologischen Interventionen (URS) an 51 Patienten (39,2 % Frauen; 60,8 % Männer; mittleres Alter 57,7 Jahre; Range 23–85 Jahre) die Hand- sowie die Kopf- bzw. Augenlinsendosis bestimmt. Durchschnittlich betrugen BMI 28,9 kg/m^2^ (minimal 19,7 kg/m^2^ resp. 48 kg; maximal 41,1 kg/m^2^) resp. KG 80 kg (minimal 48 kg; maximal 114 kg), wobei 41 % Präadipositas, 28 % Adipositas Grad 1; 6 % Adipositas Grad 2 und 2 % Adipositas Grad 3 aufwiesen.

Die transurethralen URS erfolgten in 66 % zur Steinsanierung im Rahmen einer Urolithiasis des oberen Harntraktes und in 24 % zur Diagnostik und teilweise endourologischen Ablation bei Urotheltumoren (Urothelkarzinomen) des oberen Harntrakts. Die mediane Interventionszeit betrug 65 min. Die Operateurslevel waren 45 % Level 1, 19 % Level 2 und 36 % Level 3. Das DFP betrug im Median 243,5 (minimal 21,7; maximal 3731,2) Gy/cm^2^. Die mittlere Hand- und Kopfdosis betrugen jeweils 0,05 (median 0,03) mSv. Bei beiden Messorten zeigte sich ein großer Streuungsbereich (SD Hand 0,07 resp. Kopf 0,06 mSv; minimal 0 mSv; maximal Hand 0,34 resp. Kopf 0,29 mSv). Ein höheres DFP korrelierte signifikant mit einer höheren Kopf- und Handdosis (*p* = 0,01). Ein höheres DFP und eine höhere Handdosis korrelierten signifikant mit einem höheren BMI (*p* = 0,006 bzw. *p* = 0,008) bei einer exponentiellen Korrelation zwischen dem BMI und der Kopfdosis bzw. Handdosis (Abb. 3).

Die Operationsdauer zeigte einen signifikanten Zusammenhang zum DFP, sowie einen linearen, jedoch nicht signifikanten Zusammenhang mit der Kopfdosis. Die Korrelation zwischen Erfahrungslevel der Operateure und dem DFP respektive der Kopfdosis zeigte keinen signifikanten Unterschied.

## Diskussion

Es zeigte sich eine durchschnittliche Kopfdosis von 0,05 mSv pro Intervention (maximal 0,29 mSv) bei einem durchschnittlichen DFP von 485,33 (21,7–3731,2) Gy/cm^2^ unter Verwendung moderner urologischer Röntgentischanlagen und ohne spezielle Strahlenschutzmaßnahmen für den Kopf im Sinne einer routinemäßigen Anwendung. Im Vergleich zu anderen urologischen Studien können trotz technischer und designbedingter Unterschiede die durchschnittlich hohe Augenlinsendosis bestätigt werden. Hartmann et al. maßen per TLD eine durchschnittliche Augenlinsendosis von 0,034 mSv pro URS [7]. Ritter et al. fanden per TLD ohne gepulste Durchleuchtung eine durchschnittliche Augenlinsendosis von 0,1 mSv pro URS [5]. Hristova-Popova et al. wiesen eine kumulative Augenlinsendosis am Phantom von 0,9 mSv ohne Strahlenschutzmaßnahmen und 0,06 mSv unter Verwendung eines Bleischirms, sowie in der klinischen Routineanwendung von 0,043 mSv pro URS unter Verwendung eines elektronischen EDD-30-Dosimeters nach [15]. Galonnier et al. konnten eine jährliche Augenlinsendosis von 0,31–2,3 mSv pro Jahr nach URS-Äquivalenzdosen unter Verwendung eines C‑Bogens mit entsprechend technisch bedingt geringerer Streustrahlung berechnen [6]. Bei dem Großteil der verfügbaren Studien [5, 8, 12, 15] wurden kumulierte Strahlenwerte ohne Aussagen zur durchschnittlichen und maximalen Strahlenbelastung pro Intervention erfasst. In unserer Untersuchung entsprach die maximale Augenlinsendosis pro Intervention mit 0,29 mSv dem 6‑Fachen des Durchschnittswertes. Bei Hartmann et al. mit 0,761 mSv sogar dem 38-Fachen des angegebenen Durchschnittswertes [7]. Zöller et al. untersuchten die ALD unter Verwendung eines Strahlenschutzvisiers im Rahmen der URS mit Bestimmung der Strahlendosis vor dem und hinter dem Visier. Aus der kumulativen Dosis von 12 Wochen wurde auf eine Jahresdosis berechnet. Vor dem Strahlenschutzvisier betrug die berechnete kumulative Jahresdosis 4,090 mSv und hinter dem Visier 1,737 mSv, demzufolge das Strahlenschutzvisier eine Dosisreduktion für die Augenlinse um mindestens 42 % bewirkte [4].

Das DFP gibt als Parameter zur Quantifizierung der Strahlenbelastung die kumulative Dosis innerhalb einer exponierten Fläche über die Zeit der Untersuchung an. Innerhalb dieser Fläche befindet sich der Patient bei URS in Steinschnittlage. Die auf den Patienten abgegebene Strahlung wird zu 80 % von ihm in Form von Streustrahlung reflektiert [16] und trifft auf den zwischen den Beinen des Patienten befindlichen Operateur. Das DFP weist wiederum multifaktorielle Einflussfaktoren wie den BMI des Patienten und die Operationskomplexität auf. Referenzwerte für das DFP im Rahmen einer URS existieren nicht.

Moderne Röntgenanlagen regeln den Röhrenstrom in Abhängigkeit der Patientengeometrie automatisch. Je höher der BMI des Patienten, desto größer ist der Patientendurchmesser, und desto größer ist die Strahlenabschwächung [16]. Die für eine scharfe Abbildung nun notwendige höhere Strahlenhärte bewirkt eine höhere Strahlendosis und damit auch verbundene höhere Streustrahlendosis für den Operateur [17]. Weltweit ist eine steigende Inzidenz von Adipositas zu verzeichnen, welche ein Risikofaktor für Nephrolithiasis und für den oberen Harntrakt kompromittierende Erkrankungen bzw. Behandlungen darstellt [18, 19]. In Zukunft werden die uroradiologisch-interventionspflichtige Patienten einen häufiger höheren und durchschnittlich höheren BMI aufweisen. In Folge wird auch die Strahlenbelastung des Operateurs steigen [20].

Ein weiterer möglicher Einflussfaktor stellt die Erfahrung des Operateurs, die Komplexität des Eingriffs und die damit verbundene Strahlenfeldgröße und Operationsdauer dar. Ritter et al. stellten eine signifikante Korrelation zwischen einer größeren Operateurserfahrung und einer geringeren Fluoroskopiezeit [5, 21] dar. Im Gegensatz dazu wies bei Hartmann et al. die Gruppe der erfahreneren Urologen ein höheres DFP und eine längere Fluoroskopiezeit als bei unerfahreneren auf, wobei die Augenlinsendosis bei beiden Gruppen nahezu identisch war [7]. In unserer Untersuchung zeigte sich jedoch keine signifikante Korrelation zwischen dem Erfahrungslevel des Operateurs und dem DFP. Es zeigten sich aber in der Gruppe der unerfahrensten Operateure deutlich stärkere interindividuelle Unterschiede in den Strahlendosen. Dies ist unserer Meinung nach damit zu erklären, dass die Operateure in dieser Gruppe erst am Anfang der endourologischen Ausbildung stehen und mehr Durchleuchtungen zur Orientierung benötigen. Der kleinste Streubereich mit der geringsten Standardabweichung zeigt sich dementsprechend bei den Operateuren mit >6 Jahren Erfahrung. Hierbei muss beachtet werden, dass die Operateure in Level 2 deutlich komplexere Eingriffe im Vergleich zu denen mit Erfahrungslevel 1 durchführen. Es handelt sich hierbei um Eingriffe mit hoher Steinlast, komplexen Steinlokalisationen, schwieriger anatomischer Verhältnisse oder multilokulären Tumorbefunden. Aufgrund dieser Komplexität sind mehrere Teilschritte und damit eine längere Durchleuchtungszeit notwendig, um sich zu orientieren. Dadurch entstehen höhere Strahlendosen. In der Gruppe der Operateure mit dem Erfahrungslevel 3 zeigten die Messwerte den kleinsten Streubereich. Hier ergeben sich also nur sehr geringe interindividuelle Unterschiede zwischen den Operateuren. Auch in dieser Gruppe wurden eher komplexere Eingriffe durchgeführt. Es zeigen sich jedoch geringere Strahlendosen als in den Gruppen mit geringerer Erfahrung. Unserer Meinung nach können sich die Operateure dieser Gruppe schneller und besser orientieren und müssen dementsprechend weniger Durchleuchtung einsetzen.

Angesichts des langjährigen Einsatzes von Röntgenstrahlung in der Urologie wurden bislang nur wenige Daten zur Strahlenbelastung des Operateurs publiziert. In Anbetracht der Zunahme der endourologischen Möglichkeiten sind weiterführende Untersuchungen dringend notwendig. Dosimetrische Größen wie die exakte Augenlinsendosis und tatsächliche Grenzdosis können technisch bedingt nicht direkt gemessen werden. Grenzwerte zur Vermeidung stochastischer Strahlenschäden menschlichen Augenlinsenepithels konnten aus tierexperimentellen Untersuchungsergebnissen nicht abgeleitet werden [24]. Dementsprechend werden diese Parameter durch mathematische Modelle näherungsweise errechnet [25]. Als Reaktion auf die Empfehlung der ICRP zur Reduktion des Jahresgrenzwertes der Augenlinse sprachen sich einige Experten für einen deutlich höheren Grenzwert aus [26–28].

Ein Problem bezüglich Strahlenschutzmaßnahmen stellt die Incompliance der Operateure dar. Nach einer Befragung durch Galonnier et al. [6] trugen 16 % keine Bleischürze und 86 % ihr Ganzkörperdosimeter nicht oder nur unregelmäßig während Interventionen. Ein Fingerringdosimeter wurde lediglich von 1 % der Operateure getragen [6]. Selbstverständlich kann diese Compliance nicht für alle Operateure pauschalisiert werden. Es zeigt jedoch die Notwendigkeit der besseren Aufklärung über die Folgen von unzureichendem Strahlenschutz für den Operateur. In Deutschland müssen daher alle fünf Jahre die Strahlenschutzkenntnisse aktualisiert werden. Im OP-Team sollte gemeinsam auf das ALARA(„as low as reasonably achievable“)-Prinzip zur Dosisreduktion für jeden Eingriff bewusst geachtet werden. Ein postoperatives Feedback im Sinne eines Debriefing zur Strahlenfeldeinblendung und zum DFP sind dafür insbesondere in der Ausbildungsphase hilfreich.

Weiterhin sind technische Verbesserungen notwendig. Durch den begrenzten Platz im sterilen Arbeitsbereich können bauliche Strahlenschutzmaßnahmen wie mobile Bleiglasschilde insbesondere bei der Steinschnittlage nicht bzw. kaum eingesetzt werden. Strahlenschutzvisiere sind nicht arbeitsmedizinisch bzw. strahlenschutztechnisch gefordert und nicht flächendeckend etabliert, da sie in der Regel aus Komfortgründen (Sicht, Gewicht, Akustik) vom Operateur abgelehnt werden [4]. Strahlenschutzvisiere mit einer Stirn- bzw. Kopfbefestigung sind aufgrund ihres hohen Gewichts unkomfortabel und können insbesondere zur Belastung der Halswirbelsäule führen [4]. Zusätzlich kann die Sicht aufgrund verschiedener Reflektionen ggf. noch bei zusätzlicher Laserschutzbrille eingeschränkt sein. Außerdem verschlechtert sich die Akustik und erschwert die Kommunikation mit dem Assistenzpersonal bei noch zusätzlicher Atemschutzmaske.

Röntgenschutzbrillen sind in der Regel vorhanden, werden aber auch oft aus Komfortgründen (Sicht, Gewicht) und fehlender Sensibilisierung des Personals nicht getragen. Röntgenschutzbrillen neigen eher dazu, zu beschlagen. Der Tragekomfort ist aufgrund des relativen hohen Gewichts für eine Brille bei längeren Interventionen aufgrund des Gewichts auf der Nase ebenfalls gering. Erschwert wird die Anwendung für Brillenträger mit Visuskorrektur trotz Röntgenschutzbrillen für Brillenträger. Außerdem kommt es auch hier je nach Modell zu störenden Lichtreflektionen. Weiterhin kommen verschiedene Laser bei einem Großteil der endourologischen Eingriffe (Laserlithotripsien und Lasertumordestruktionen im oberen Harntrakt) unter fluoroskopischer Kontrolle zur Anwendung, wobei Kombinationen aus verschiedenen Laserschutzbrillen und Röntgenschutzbrille nicht zur Verfügung stehen.

Zusammenfassend zeigten sich bei den transurethralen uroradiologischen Interventionen (Ureterorenoskopien) teils sehr hohe Einzeldosen und durchschnittlich hohe Einzeldosen bzw. eine durchschnittlich hohe kumulative Dosis für die Augenlinse der Operateure. Insbesondere Urologen und Urologinnen mit einem endourologischen Schwerpunkt für uroradiologische Interventionen ab der intensiveren Weiterbildungsphase zum Facharzt und darüber hinaus sind von der höheren Strahlenexposition betroffen. Mit lediglich 400 Eingriffen pro Jahr oder durchschnittlich lediglich 2 Eingriffen pro Arbeitstag wäre nach unseren Daten damit der kritische Jahresgrenzwert für die Augenlinsen bzw. für das Risiko eines Strahlenkatarakts ohne spezielle Schutzmaßnahmen in unserem Setting überschritten. Dies erscheint in der Krankenhausroutine eine problemlos erreichbare Größe für interventionell spezialisierte Urologen, sodass ein konsequenter, effektiver Strahlenschutz der Augenlinse essentiell für die tägliche Arbeit ist. Hierfür sind ggf. technische Weiterentwicklungen für eine komfortablere und einfachere Anwendung erforderlich.

## Limitationen

Das Fading der verwendeten TLD beträgt maximal 10 % pro Jahr bei Raumtemperatur und ist somit für den Studienzeitraum vernachlässigbar. Die Teilkörperdosimetrie der Kopfoberfläche auf geeichte 0,07 mm mittels TLD stellt eine Näherungsweise zur Erfassung der Augenlinsendosis gemäß der deutschen Strahlenschutzkommission dar [22, 23]. Eine damit einhergehende leichte Dosisüberschätzung der mit 3 mm tiefer gelegenen Augenlinse ist jedoch im Sinne der Grenzwertdosisüberwachung zielführend. Die Eingriffe wurden nicht nach Komplexität, Durchleuchtungszeit und Röntgenfeldgröße unterteilt. Ebenso wurden keine individuellen Daten zur Statur und exakten Kopfposition des Operateurs erfasst. Die Kopfposition des Operateurs bei transurethralen Eingriffen in Steinschnittlage ist jedoch ausgerichtet an der Arbeitshöhe nur in einem geringen Bereich innerhalb des Streustrahlenfelds variabel.

## Fazit für die Praxis

- Im Rahmen von interventionell-uroradiologischen Interventionen sind die Operateure insbesondere bei transurethralen Eingriffen in Steinschnittlage einer hohen Streustrahlenbelastung ausgesetzt.
- Bei einer hohen Eingriffszahl und -frequenz sowie langen Berufstätigkeit kann eine relevante Augenlinsendosis akkumulieren und die jährliche Grenzdosis von 20 mSv überschritten werden.
- Neben dem ALARA(„as low as reasonably achievable“)-Prinzip zur Dosisreduktion sollte Strahlenschutzmaßnahmen für die Augen genutzt werden, um einen Strahlenkatarakt zu vermeiden.
- Für eine höhere Anwendercompliance sind technische Weiterentwicklungen notwendig.
